# How Organizational Accommodation Converts Continuity of Care into Equitable Patient Experience and Satisfaction

**DOI:** 10.3390/healthcare14213564

**Published:** 2026-10-22

**Authors:** Scott A. Phillips

**Affiliations:** Healthcare Management Program, Louisiana State University New Orleans, New Orleans, LA 70148, USA; sphilli2@lsuneworleans.edu

**Keywords:** patient satisfaction, patient experience, quality of health services, patient-centered care, continuity of care, health equity, healthcare management

## Abstract

Background/Objectives: Continuity of care, patient experience, and satisfaction are central concerns of healthcare management, yet continuity arrangements may transfer coordination work to patients whose resources differ substantially. This conceptual study develops a model explaining when continuity supports patient-centered care and how managers can evaluate its equity implications. Methods: A purposive, theory-guided synthesis integrated Donabedian’s structure–process–outcome framework with Burden of Treatment Theory and expectation–disconfirmation reasoning. Foundational publications, recent empirical studies, and measurement research informed construct boundaries, directional relationships, and a prospective evaluation design. References were checked against DOI registration records. No primary participant data were collected or analyzed. Results: The proposed Continuity Conversion Model identifies navigation burden as an intervening mechanism connecting organizational continuity with patient experience and subsequent satisfaction. Organizational accommodation, defined as accessible support matched to patients’ practical needs, is proposed to strengthen continuity’s association with lower burden. Four hypotheses specify these relationships; two propositions address organizational remediation and equity monitoring. Operationalization tables distinguish audited processes, patient-reported constructs, contextual characteristics, and clinical balancing outcomes. Two flowcharts depict the theoretical pathway and a feedback process for repairing service failures. A longitudinal design separates measurement occasions and permits tests of conditional indirect associations, competing explanations, and subgroup differences. Conclusions: Continuity should be assessed by whether patients can use connected services without excessive navigation work. The model offers a testable management approach that treats equity as a distributional question and satisfaction as an evaluative outcome rather than a sufficient indicator of quality. Its explanatory value and proposed measures require empirical validation.

## 1. Introduction

Patient experience and patient satisfaction answer related but different questions. Experience concerns what occurs during care, including communication, involvement, and respectful treatment; satisfaction evaluates care against expectations and other personal standards [1]. Their distinction matters when managers interpret performance scores. A pleasant encounter may receive favorable ratings while leaving an unresolved referral, unaffordable treatment, or inaccessible follow-up. Conversely, a technically appropriate decision may disappoint a patient. A review of satisfaction research identified multiple determinants and substantial measurement diversity [2]. Quality assessment therefore needs an explanation of how service arrangements become usable care, rather than assuming that higher ratings represent improvement throughout the care pathway.

Patient experience remains a substantive quality domain. Doyle and colleagues found predominantly positive associations between experience, safety, and effectiveness across heterogeneous studies, although associations do not establish a universal causal relationship [3]. Continuity provides one plausible organizational route to better experience. Its informational, management, and relational dimensions concern the availability of relevant knowledge, coherent care arrangements, and sustained therapeutic relationships [4]. However, records can travel successfully while patients repeatedly telephone offices, reconstruct instructions, or negotiate conflicting appointments. Documented coordination and lived continuity may diverge when the patient becomes the unpaid integrator of services.

This divergence has an equity dimension. In a United States survey of 4155 insured adults aged 18–64, Kyle and Frakt found that 24.4% reported delayed or forgone care associated with administrative tasks; disability and income were relevant to the distribution of burden [5]. These findings concern a particular population and period, but demonstrate that administrative work can obstruct access even among insured patients. More recently, Browne and colleagues developed equity-oriented experience measures that explicitly examine how care responds to structurally disadvantaged populations [6]. Together, these studies motivate examining whether continuity benefits depend on practical organizational support.

This article proposes the Continuity Conversion Model for adults with chronic conditions moving between hospital and ambulatory services. Its central question is: under what organizational conditions does continuity reduce navigation burden and improve patient experience and satisfaction? The contribution is a specified mechanism and boundary condition, supported by four hypotheses, two organizational propositions, and a measurement architecture. The model connects established ideas rather than claiming to discover continuity, treatment burden, or equitable care. Its proposed novelty lies in treating organizational accommodation as a condition governing the conversion of continuity arrangements into usable patient-centered care.

The four hypotheses are stated below to make the aims explicit. They are proposed for future testing and are not tested in this conceptual article. Section 3.2 supplies their rationale and two additional organizational propositions.

**H1.**  *Greater audited continuity during the transition episode will be associated with lower subsequent patient navigation burden, conditional on baseline clinical complexity and navigation resources.*

**H2.**  *Greater navigation burden will be associated with poorer subsequent patient experience, after accounting for earlier experience and clinical complexity.*

**H3.**  *Better patient experience will be associated with greater subsequent patient satisfaction, after accounting for baseline expectations, prior satisfaction, and clinical status.*

**H4.**  *Greater organizational accommodation will strengthen the negative association between continuity and navigation burden, such that the continuity-burden slope is more negative where accommodation is greater, primarily among patients with at least one baseline navigation constraint.*

## 2. Materials and Methods

### 2.1. Conceptual Design and Evidence Selection

This study combines published evidence and established theories to propose an explanation that future studies can test using theory synthesis and model development [7]. The original manuscript reports targeted web searches of scholarly publisher pages and PubMed-indexed records on 14 September 2026, with backward reference checking. Its reported topics were patient experience and satisfaction; continuity of care and patient burden; patient administrative burden; equity-oriented care; and continuity measurement. Selection prioritized explanatory relevance, primary empirical studies, original theoretical contributions, and measurement development. Recent publications supplemented foundational work. Retrieval was purposive and limited to English-language material; no fixed publication-year cutoff was reported. This was not an exhaustive database review and did not use duplicate screening, pooled effects, or a systematic-review protocol.

The available record of the original search does not include exact queries, retrieval counts, or a contemporaneous screening log. To improve the auditability of this revision, supplementary targeted web searches were conducted on 12 October 2026 using the OpenAI web search interface (OpenAI, San Francisco, CA, USA). Appendix A, Table A1 records the exact query strings and the primary source anchors checked. These searches verify sources and clarify concepts already cited; they do not constitute an exhaustive update or reproduce the historical result set. No date filters were applied to these supplementary queries. Scholarly publisher and PubMed records were preferred for verification; other web results and this manuscript’s own preprint were not used as independent support. Search ranking and availability may change, so the query record is reproducible as a procedure rather than as an identical result set.

The evidence was used to compare definitions, distinguish reported associations from proposed mechanisms, and consider alternative explanations. DOI registration metadata were used in the original manuscript to check publication identity; accessible publisher or indexed records supported interpretation. The revision also checked relevant primary publisher and PubMed records. Bibliographic verification confirms source identity, not the validity of every claim or a full-text appraisal of every publication. The resulting framework is an analytical proposal rather than evidence of causal pathways. No participant observations or statistical results were generated or analyzed.

ChatGPT (GPT-6.1 Sol Ultra; OpenAI, San Francisco, CA, USA) assisted with literature discovery, conceptual development, drafting, and figure preparation, as disclosed in the original Acknowledgments. The documented discovery role was assistance in identifying potentially relevant literature for source checking. AI-generated suggestions were not themselves treated as scholarly evidence. The original query and prompt history are not documented in the materials available for this revision; it is therefore not possible to specify whether original discovery included particular keyword-generation, query-expansion, summarization, or screening operations. No automated screening procedure or AI-derived evidence extraction is claimed. The date 14 September 2026 is the reported search/access date, not a documented one-day rapid-review or analysis protocol. ChatGPT also assisted with the supplementary source searches, explanatory revisions, and figure programming on 12 October 2026. The author remains responsible for selection, interpretation, accuracy, and final approval.

### 2.2. Model Development in Plain Language

The six steps below summarize the logic used to develop the model. They describe how the concepts lead to the proposed outputs; they are not a historical screening or task log.

1. Define the problem. Ask why apparently connected care can remain difficult to use. Separate what happened during care from how patients evaluated it. This establishes the question about continuity, navigation work, and usable support.
2. Locate relevant evidence. Use targeted literature discovery and reference checking to find publications explaining the concepts, their possible relationships, and their measurement. This creates a purposive evidence base rather than an exhaustive review.
3. Compare the concepts. Compare published definitions and decide what belongs inside each construct. Separate navigation tasks from symptoms, resources, encounter experience, and satisfaction to reduce overlap.
4. Connect the ideas. Use quality assessment to locate structures, processes, and outcomes; use workload and capacity to explain patient work; and use expectations to explain satisfaction. Consider alternative explanations before specifying the pathways.
5. State predictions. Translate the proposed relationships into H1–H4 and P1–P2. Identify findings that would contradict the predictions so that later studies can challenge the explanation.
6. Plan empirical evaluation. Specify candidate indicators, timing, comparison conditions, and subgroup reporting. These are future measurement and testing plans; no participant observations or statistical tests are reported here.

### 2.3. Theoretical Integration

Donabedian’s framework distinguishes organizational structures, care processes, and outcomes [8]. Here, support infrastructure is structural, continuity is a care process, and experience and satisfaction are distinct patient-reported outcomes. The framework organizes assessment but does not itself explain why apparently similar processes yield unequal experiences. Burden of Treatment Theory explains the work people must do to manage care: people undertake healthcare work using resources embedded in their personal and social circumstances [9]. The cumulative complexity model asks whether care demands exceed the time, energy, money, and support available to meet them [10]. Navigation burden narrows this reasoning to the difficulty imposed by obtaining, connecting, and sustaining care across organizational boundaries.

Patient-centeredness provides the normative orientation. Scholl and colleagues’ integrative model includes communication, involvement, access, and continuity within a broader set of dimensions [11]. Organizational accommodation is defined here as the availability of usable support matched to identified navigation needs, including language assistance, scheduling flexibility, transport coordination, and nondigital access. It is neither patient motivation nor general organizational friendliness. Expectation–disconfirmation reasoning describes how people compare what happened with what they expected. It explains why the same experience need not produce the same satisfaction: evaluations reflect prior standards as well as perceived performance [12]. Expectations are consequently measured before the episode and treated as an explanatory covariate, without assuming that lowering expectations constitutes improvement.

This integration also allows direct pathways. Continuity may improve reassurance through a familiar relationship even when navigation work does not change, and accommodation may reduce burden without improving continuity. The proposed sequence is therefore partial rather than exclusive mediation. Its distinctive prediction is that combining continuity with usable support yields an additional benefit beyond their separate associations. A null interaction with precisely estimated additive benefits would support investment in both services, while rejecting the model’s complementarity claim. The framework consequently separates a useful management intervention from confirmation of a particular theoretical explanation.

### 2.4. Scope and Model Construction

The unit of explanation is the patient’s transition episode, nested within a receiving care organization. The initial application concerns adults with at least one chronic condition discharged home with planned ambulatory follow-up. Patients whose follow-up never occurs remain eligible because exclusion would remove an important failure pathway. The conceptual sequence separates organizational provision, patient navigation work, experienced interactions, and subsequent evaluation. The proposed measurement plan reduces construct overlap by specifying independent continuity audits and excluding continuity and scheduling items from the primary experience measure. The model does not presume that satisfaction is a surrogate for safety, clinical effectiveness, or population health equity.

## 3. Results

### 3.1. The Continuity Conversion Model

The conceptual result is a conditional pathway linking continuity, navigation burden, patient experience, and satisfaction, with organizational accommodation modifying the first relationship. Figure 1 displays the four hypothesized paths. Continuity should reduce repeated information gathering and uncertainty about responsibility. Its benefit may nevertheless be limited when an accessible next step is unavailable. A familiar team and complete discharge record provide little practical relief if the patient cannot obtain interpretation or contact the receiving service through an accessible channel. Accommodation is proposed to make continuity arrangements actionable under such conditions.

A mediator is a proposed intervening step: here, navigation burden could help explain how continuity relates to later experience. A moderator changes the strength of a relationship: here, accommodation could change how much continuity reduces burden. Figure 1 traces the serial indirect pathway C → B → E → S and shows additional direct terms to avoid assuming that burden is the only route to better experience. Its arrows are theoretical predictions, not relationships found in participant data.

The central boundary condition is complementarity between continuity and accommodation. This differs from treating patient resources as reasons to expect weaker performance. Resources describe the circumstances under which care must function; accommodation describes an organizational response. A 2025 review of Burden of Treatment Theory supports its relevance across settings while identifying the need for further adaptation and critical assessment [13]. Accordingly, the proposed interaction is falsifiable: if accommodation substitutes for continuity, its benefit may be largest when continuity is poor, producing the opposite interaction. Either result would inform service design, but only the predicted complementary pattern would support the present formulation.

Existing continuity models already recognize personalized care, contextual resources, and interdependent service relationships. Ljungholm and colleagues’ grounded theory model emphasizes timely care and coordination across time and space [14]. The present extension specifies navigation burden as an intervening variable, separates satisfaction from experience, and locates accommodation at an explicit interaction point. It also requires examining the distribution of gains. The term continuity conversion denotes this proposed explanatory process; it is not a validated scale, a mathematical efficiency ratio, or an assertion that earlier continuity research ignored patients’ circumstances.

### 3.2. Hypotheses and Organizational Propositions

**H1.**  *Greater audited continuity during the transition episode will be associated with lower subsequent patient navigation burden, conditional on baseline clinical complexity and navigation resources.*

The rationale is that clear responsibility and usable shared information reduce the patient’s need to reconstruct the care pathway. A scoping review describes continuity and coordination as related components of primary healthcare, with organizational and system conditions shaping implementation [15]. Recent English practice-level research also links continuity with complaints, but its repeated cross-sectional design cannot establish the individual mechanism proposed here [16]. H1 therefore concerns a prospectively observed association rather than an assumed intervention effect.

**H2.**  *Greater navigation burden will be associated with poorer subsequent patient experience, after accounting for earlier experience and clinical complexity.*

Burden can consume time and attention needed for participation, increase uncertainty, and force patients to repeat unmet needs. This prediction concerns subsequent communication, respect, and involvement, not dissatisfaction relabeled as burden. Navigation questions must therefore describe work and difficulty, while experience questions describe interactions. A patient may report substantial work alongside respectful care; such observations are informative exceptions rather than measurement errors.

**H3.**  *Better patient experience will be associated with greater subsequent patient satisfaction, after accounting for baseline expectations, prior satisfaction, and clinical status.*

The expected positive association preserves the conceptual distinction between events and their evaluation. Expectations may explain why equivalent experiences generate different ratings. The model does not require complete agreement between measures. In particular, persistently high satisfaction alongside poor experience should prompt examination of response patterns, dependence on services, and restricted alternatives rather than automatic acceptance of a quality claim.

**H4.**  *Greater organizational accommodation will strengthen the negative association between continuity and navigation burden, such that the continuity-burden slope is more negative where accommodation is greater, primarily among patients with at least one baseline navigation constraint.*

Accommodation is assessed before follow-up, independently of whether the patient subsequently reports success. The prediction is most relevant when practical barriers exist and continuity can connect patients to available support. Services should retain a direct accommodation-to-burden term when testing the interaction; otherwise, a general support benefit could be incorrectly attributed to complementarity. The corresponding estimands and disconfirming findings are specified in Section 3.4.

**P1.**  *Among organizations with comparable continuity processes, a remediation process that assigns responsibility and verifies resolution with patients will produce larger reductions in recurrent navigation failures than complaint acknowledgment without verified resolution.*

**P2.**  *When accommodation resources are allocated according to identified navigation needs, improvements in continuity will produce larger reductions in experience gaps between patients with greater and fewer navigation constraints than uniformly distributed support of equivalent total cost.*

The propositions address organization-level policies and require comparative evaluation. P2 predicts distributional improvement rather than universal convergence or equal outcomes. It permits failure when need identification is inaccurate, support is inaccessible, or allocation stigmatizes intended beneficiaries. Neither proposition adds a second interpretation of the patient-level hypotheses.

### 3.3. Operationalization and Measurement Boundaries

Table 1 distinguishes constructs, indicators, data sources, and scoring. Audited continuity is a proposed formative index, meaning that different components jointly define the construct: informational, management, and relational components contribute different content and need not correlate strongly. Accommodation is also formative because interpreters, scheduling options, and accessible contact channels are not interchangeable manifestations of one psychological trait. Because distinct services need not correlate, agreement among questionnaire items alone cannot establish validity for either index. Prespecified scoring, content review, inter-rater agreement, and sensitivity to alternative weights are more relevant. Reporting the component scores alongside totals prevents strong performance in one domain from concealing an absent essential service.

Accommodation scoring should match provision to needs identified at baseline. For each applicable support domain, an auditor would verify an accessible service, a responsible contact, and availability within the required timeframe. The proportion of domains meeting these criteria forms the proposed patient-specific score, nested within the organization. No identified need is coded separately, and none awarded a perfect accommodation score. H4 is primarily evaluated among patients reporting at least one navigation constraint; H1–H3 retain the broader eligible cohort. Actual uptake and later service failures are recorded separately to avoid defining the moderator by its downstream success.

Navigation burden is the time, effort, and difficulty involved in obtaining and connecting needed services. It includes arranging appointments, locating the responsible service, obtaining information required to proceed, completing referral or approval steps, and reconciling instructions across providers. It is narrower than general treatment burden, which also includes taking medicines, monitoring symptoms, and making recommended lifestyle changes [9,18]. Administrative burden overlaps when paperwork or organizational rules complicate access; navigation additionally concerns connecting clinical instructions and responsibilities across services [5]. These are analytical distinctions, not claims that the domains never overlap.

Illness severity and symptoms may make navigation harder, but are not themselves navigation tasks. Financial strain, language, digital access, transport, and available help describe constraints and resources. Their presence should not automatically add points to the burden score. The work required to address them can be measured separately. For example, difficulty using a portal is a constraint, repeated attempts to reach a clinic are navigation work, and an accessible alternative channel is accommodation. Respect and communication during the eventual encounter are experience; the overall evaluation of care is satisfaction. The same incident may affect several constructs, but each measure must ask a different question.

Navigation tasks are not automatically unnecessary. A diary should record the activity, who performed it, the time spent, perceived difficulty, and whether help was wanted. Clinically necessary work and potentially avoidable repetition should be distinguished during analysis rather than excluded by definition. Patient and caregiver work should remain separate, and dissatisfaction wording should be excluded from navigation items. A patient can therefore report substantial navigation work alongside respectful care and high satisfaction without an inconsistency in measurement.

Navigation burden is measured separately from general treatment burden. The Multimorbidity Treatment Burden Questionnaire provides an established comparator, including subsequent development work, but changing its content or recall period creates an adaptation requiring validation [18,19]. Likewise, the Patient Experienced Continuity of care Questionnaire can provide convergent evidence for audited continuity, without being reused as the experience outcome [17]. The proposed navigation items are original candidates. Cognitive interviews should establish whether patients distinguish unnecessary navigation from clinically necessary tasks, and whether response options work across languages, disability accommodations, and educational backgrounds.

The primary experience outcome is a prespecified composite of communication, respect, and preference involvement domains, with original candidate items and separate domain reporting. It excludes satisfaction wording and administrative continuity content. Satisfaction is a distinct global evaluation. Equity is assessed through distributions of burden, experience, and completed follow-up across prespecified social groups and navigation constraints, not through a single patient-level equity score. Research relating social drivers to experience supports this distributional emphasis, while remaining observational [20]. Clinical safety, unmet need, and staff workload are balancing outcomes because favorable ratings alone cannot establish the quality of health services.

Failure to receive care requires special handling. Patients without an opportunity to assess an interaction should not be forced to assign a respect or communication rating. A two-part analysis would first examine whether needed contact occurred and then examine experience conditional on contact. The conditional experience estimand must be labeled explicitly, with selection sensitivity analyses and separate reporting of access failures. Structural absence of an interaction is different from an unanswered survey item. Treating either as a neutral midpoint would obscure barriers and could make an organization appear better by excluding its least successfully connected patients.

### 3.4. Prospective Evaluation and Remedial Workflow

A prospective multisite cohort could assess accommodation and baseline characteristics at discharge, continuity over days 1–7, navigation burden over days 8–14, experience at day 21, and satisfaction at day 30. Repeated baseline and follow-up measurements would improve separation of stable reporting tendencies from episode-specific change. Interview and paper options would supplement digital collection. Analyses would account for clustering, baseline morbidity, prior utilization, insurance, navigation resources, and previously reported experience and satisfaction. Measures and contact procedures should include participants whose follow-up fails, using minimal-burden outreach and recording reasons for nonresponse. Table 2 summarizes the proposed estimands and disconfirming evidence.

The initial analysis would estimate multilevel path models with direct continuity and accommodation terms, their interaction, and the subsequent burden–experience and experience–satisfaction relationships. The conditional indirect association is the product of the continuity-burden slope at a specified accommodation level and the two downstream slopes. It is derived from H1–H4 rather than introduced as another hypothesis. Uncertainty should reflect clustering and uncertainty throughout the path. Causal mediation requires stronger assumptions, including adequate control of exposure–mediator and mediator–outcome confounding; temporal ordering alone does not satisfy them [22]. Observational estimates would be reported accordingly.

In plain language, the proposed analysis asks how much continuity relates to lower burden at different levels of accommodation, then traces the association through later experience and satisfaction. This product of successive relationships is a conditional indirect association. Direct continuity-to-experience and accommodation-to-burden terms remain in the model so that the indirect pathway is not forced to explain all associations. A causal interpretation would need additional evidence beyond this prospective ordering.

Inference should emphasize estimated magnitudes and intervals. Continuity and accommodation scores would be centered before forming their product, and marginal continuity slopes would be reported at substantively interpretable accommodation levels. A prespecified smallest effect of interest would distinguish an inconclusive estimate from evidence against a meaningful association. Models would test domain-specific continuity effects and alternative index weights. Missing covariates could be multiply imputed under stated assumptions, whereas nonresponse would receive separate sensitivity analysis for departures from missing at random. Selection into observed contact requires additional assumptions and cannot be repaired automatically by ordinary imputation or response weighting.

Figure 2 translates the model into a proposed remedial process. Managers first distinguish continuity failures from unmet accommodation needs, then assign an owner, repair the relevant process, and confirm resolution through the patient’s preferred channel. Recurrence triggers further review. Aggregate monitoring examines burden and experience alongside equity and safety indicators. This workflow is a design proposal and has not been tested. Urgent clinical concerns require immediate clinical escalation rather than waiting for administrative review. Resolution means that the identified obstacle has been addressed in a way the patient can use, not merely that a ticket has been closed.

## 4. Discussion

### 4.1. Theoretical Contribution and Competing Explanations

The model offers three incremental advances. First, it separates the continuity an organization provides from the navigation work a patient must still perform. Earlier continuity theory distinguishes informational, management, and relational continuity [4], while Ljungholm and colleagues explain how personalized information, clear responsibilities, cooperation, and trust work together across time and settings [14]. The present model builds on these contributions by specifying navigation burden as a possible intervening pathway. Second, it predicts that continuity will have a stronger association with lower burden when usable organizational support matches identified needs. This interaction is more specific than the claim that continuity and support are each beneficial. Third, it separates descriptions of care encounters from satisfaction judgments and examines whether benefits reach patients facing greater navigation constraints. The contribution is a testable configuration of established concepts; it does not imply that previous models ignored context or equity.

For example, sending a complete discharge record may establish informational continuity while leaving a patient unable to arrange follow-up through an inaccessible telephone or digital service. An accessible contact channel could make that information usable. This illustrates the proposed interaction, without demonstrating an effect. Direct benefits of continuity and accommodation are also permitted. Precisely estimated additive benefits would challenge complementarity while preserving the practical value of both services.

Several competing explanations remain plausible. Better-resourced organizations may independently provide continuity, accommodation, and respectful care. Patients with stronger social support may select such organizations or complete surveys more readily. Greater illness severity may simultaneously increase navigation work and worsen experience. Conversely, earlier dissatisfaction may increase perceived difficulty or prompt movement between providers, weakening continuity. Adjustment reduces some ambiguity but cannot eliminate unmeasured confounding. A future cluster-randomized factorial study of continuity support and accommodation, following feasibility work, would offer a stronger test of complementarity. Assignment would concern additional supports while preserving routine and clinically necessary services.

### 4.2. Implications for Healthcare Management

For managers, the practical question becomes which work the organization has transferred to the patient and whether that transfer is necessary. A discharge call that creates several additional calls may increase activity without reducing burden. A named coordinator with authority to solve an access problem may be more useful than repeated reminders. These are illustrative mechanisms, not established comparative effects. Budgets should capture staff time and patient navigation time separately, permitting evaluation of whether apparent organizational efficiency simply moves cost outside the organization. Patient advisory participation can identify tasks that routine administrative records overlook.

Patient-centered care also requires preserving agency. Some patients prefer to coordinate parts of their care, choose a different clinician, or decline assistance. Accommodation should respond to those preferences without classifying refusal as noncompliance. Relational continuity must therefore remain compatible with patient choice and access to appropriate expertise. Satisfaction should be interpreted alongside experience and clinical indicators: a national observational study associated higher satisfaction with several utilization and expenditure outcomes and greater mortality, illustrating the danger of equating satisfaction with clinical benefit [21]. These findings do not establish that satisfaction itself causes harm.

That study’s satisfaction composite also incorporated communication-related items, underscoring the measurement overlap this model seeks to avoid. Its findings justify caution about broad ratings, not a prediction that the proposed satisfaction measure will reproduce the same associations.

### 4.3. Applicability Across Financing and Delivery Systems

The model concerns how care becomes usable and is not restricted to one financing arrangement. Financing and delivery should be examined separately: a shared funding source need not produce shared records or clear responsibility, and an integrated provider organization need not remove practical or financial barriers. The empirical questions are who funds support, which organization can resolve a barrier, and whether the service needed after discharge is available. The following scenarios illustrate possible adaptations; they are not comparative findings about particular countries.

In a publicly financed or pooled-insurance pathway with limited patient charges, accommodation might focus on understandable referral instructions, appointment timing, language access, and transport. Where patients must secure insurer approval or move between contracted provider networks, it might also include help with authorization, coverage, and eligibility. Administrative tasks can obstruct access, as the United States study illustrates [5], but their form and frequency must be assessed locally. In an integrated network, a coordinator with authority across departments could address internal handoffs while preserving patient choice and appropriate access outside the network. Where follow-up capacity is limited, accommodation might organize realistic referral and travel options; navigation assistance cannot create an unavailable clinical service.

International applications should adapt support indicators to local responsibilities while preserving the distinction between continuity, navigation work, encounter experience, and satisfaction. Studies should document patient charges, referral and authorization requirements, service capacity, and organizational authority to act. Researchers should check whether translated items mean the same thing and whether settings offer comparable opportunities to receive care before comparing scores. Financing arrangements may influence what support is feasible and who bears its cost; they are contextual conditions to examine, not evidence that a particular system guarantees equity.

### 4.4. Equity and Implementation Safeguards

Equity analysis should identify who benefits, who remains burdened, and whose experience is absent from the data. Race and ethnicity represent social positioning and exposure to structural conditions, not intrinsic patient deficits. Language, disability, financial strain, transport, and digital access should be measured directly where relevant. Organizations should report unadjusted subgroup distributions alongside clearly justified adjusted estimates. Adjustment for circumstances produced by structural disadvantage can conceal the disparity managers need to address. Small subgroup estimates require uncertainty intervals and privacy protection; aggregation decisions should be developed with affected communities rather than imposed solely for convenient reporting.

For equity monitoring, a smaller gap is insufficient if outcomes deteriorate for everyone. Reports should show each group’s absolute experience level, its change over time, and the between-group difference. A reduction caused only by declining outcomes among previously advantaged patients would not meet the model’s improvement criterion. Similarly, unchanged average experience could conceal meaningful gains among patients previously excluded from follow-up. These distinctions make the proposed equity assessment both distributional and directional, and protect against evaluating success through a single pooled average alone across participating healthcare organizations.

Implementation should begin with one defined transition pathway and an explicit service owner. Managers can compare recurrent failures before and after a pilot while documenting concurrent changes and avoiding causal claims from simple pre–post differences. Patient confirmation, accessible reporting channels, and an appeal route make remediation observable. A worsening staff workload or safety signal should trigger redesign even when satisfaction improves. P1 and P2 ultimately require comparison conditions and adequate organizational replication. Their evaluation should include implementation fidelity, unintended stigma, and whether resources actually reach patients with the greatest navigation constraints.

### 4.5. Limitations and Research Priorities

This conceptual study cannot demonstrate effectiveness, establish causal mediation, or certify novelty across all related studies. Purposive English-language retrieval and incomplete original search documentation may miss relevant work and limit replication, and evidence from different health systems may not transfer directly to United States transitions. Several constructs are newly specified, with no established thresholds or minimally important differences. Prospective validation must address content coverage, measurement invariance, recall, ceiling effects, and discriminant validity. Sample size should be determined through simulation informed by pilot variability, clustering, attrition, and the interaction of interest. An arbitrary participant-to-item rule would provide insufficient assurance for the proposed multilevel model.

Future research should proceed in stages. First, interviews, task diaries, and cognitive testing across languages and levels of disability, digital access, and clinical complexity should establish whether patients distinguish the constructs as intended. The prospective multisite cohort in Section 3.4 can then examine timing, feasibility, competing explanations, and variation across sites. It should include patients whose planned follow-up fails. Such a cohort tests prospective associations; temporal ordering alone does not establish causality.

After feasibility work, the most direct experimental test of the proposed complementarity would be a cluster-randomized 2 × 2 factorial study with four arms: routine care; additional continuity support; additional accommodation; and both enhancements. Routine and clinically necessary services would continue in every arm. The enhancements must have distinct, documented components. On a prespecified additive burden scale, the interaction asks whether the combined reduction exceeds the sum of the two separate reductions. A combined package outperforming routine care alone would not establish complementarity. Randomizing the enhancements tests their interaction; it does not automatically validate the observational C × A scores or establish causal mediation through burden, which still requires assumptions and sensitivity analysis [22].

When randomization is impractical, controlled longitudinal evaluations could compare service changes with concurrent changes in similar organizations. These studies should document concurrent policies, case mix, implementation fidelity, and the assumption that comparison sites represent the trajectory that intervention sites would otherwise have followed. Comparative work should include rural and urban sites, different financing and delivery arrangements, and deliberate recruitment of people with substantial navigation constraints. Researchers should prespecify subgroup interactions, check measurement comparability, and use simulation to size the study for the interaction and subgroup contrasts, including adequate numbers of organizations. Access completion, caregiver work, staff workload, safety, and costs remain companion outcomes.

A further priority is distinguishing helpful delegation from avoidable work. Navigation diaries should record who performed each task, why it was required, and whether the patient wanted assistance. Otherwise, lower recorded patient effort might merely reflect greater unpaid caregiver labor. Comparative evaluations should therefore measure caregiver time and burden when consent and participation permit. Sustained improvement requires fewer unresolved obstacles across the household, not only a lower patient score. Replication across rural, urban, and differently financed systems would clarify which relationships are portable and which depend on local service capacity.

## 5. Conclusions

The Continuity Conversion Model proposes that continuity supports patient-centered care partly by reducing navigation burden, with organizational accommodation strengthening that relationship. Four hypotheses and two propositions translate the argument into testable patient-level pathways and organizational comparisons. Separating experience from satisfaction, and examining the distribution of benefits alongside clinical outcomes, provides a more accountable approach to quality assessment. Empirical research must establish whether the proposed complementarity exists, whether the measures perform adequately, and whether remediation improves care for patients facing the greatest practical barriers.

## Figures and Tables

**Figure 1:**
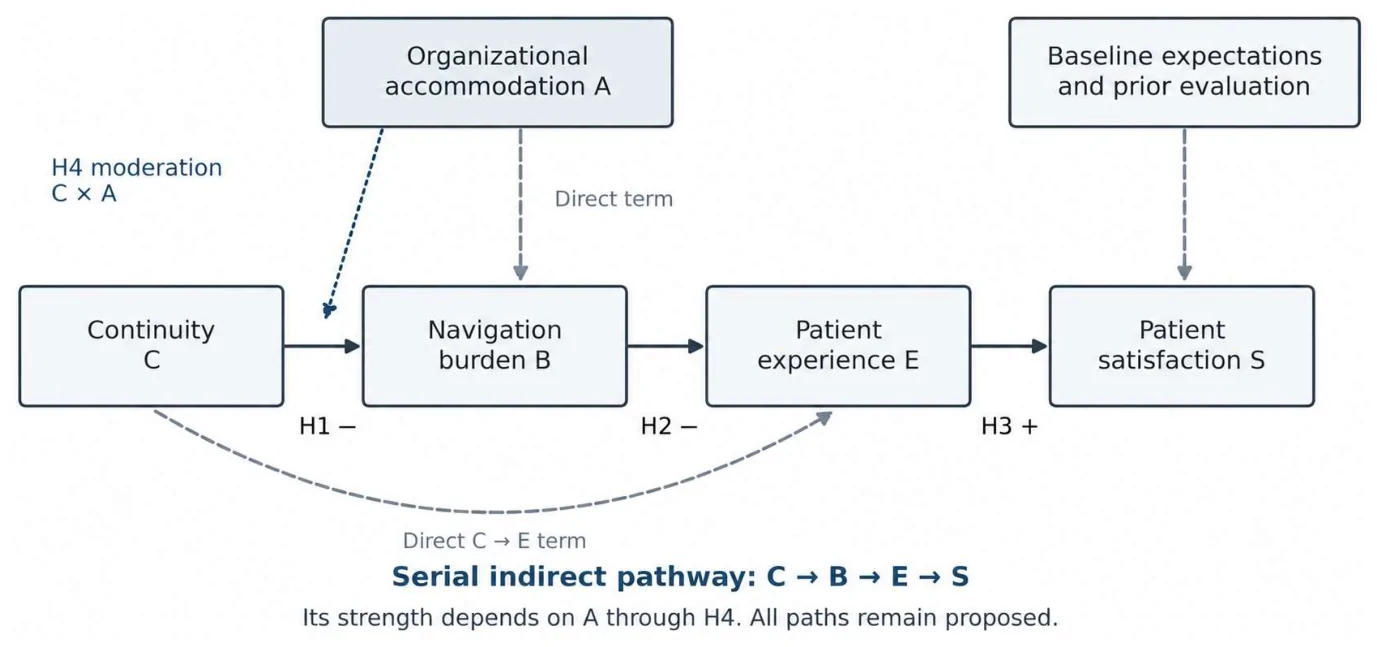
Proposed Continuity Conversion Model. Solid arrows show H1–H3. The dotted arrow shows H4 moderation of the continuity-to-burden slope among patients with baseline navigation constraints. Dashed arrows show additional direct or explanatory terms. The serial indirect pathway is traced below the diagram; it is derived from H1–H4, not another tested hypothesis. Experience is assessed conditional on contact, and access failures are reported separately. Baseline clinical complexity, resources, and other covariates are retained in analysis but omitted from the figure for readability. The assessment architecture and burden mechanism inform the model [8,9,10].

**Figure 2:**
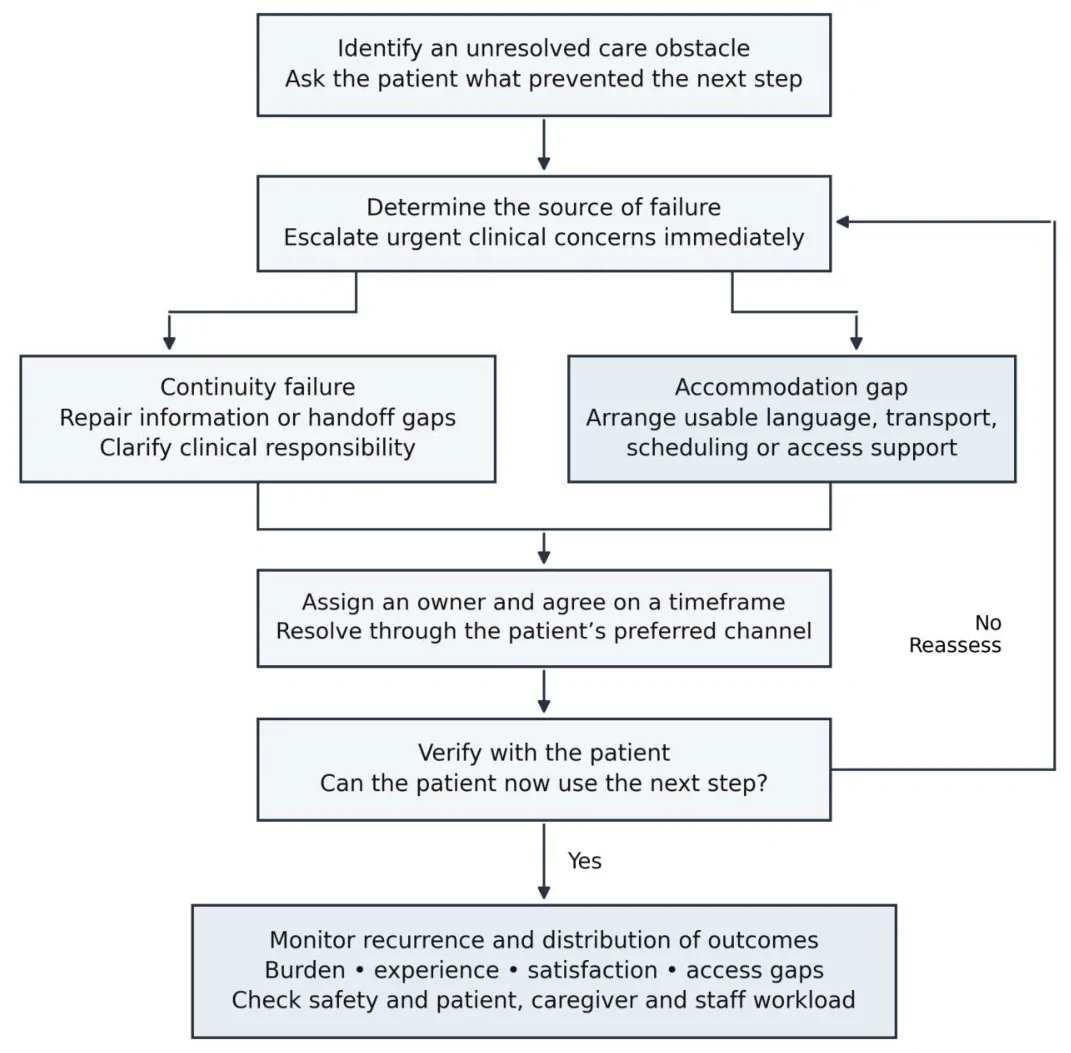
Proposed remediation of continuity and accommodation failures. Both branches may apply to one episode. Reassessment continues when the patient cannot use the next step. The workflow operationalizes P1 and P2 and has not been empirically evaluated.

**Table 1:** Operational definitions and proposed measurement plan.

Construct and Role	Proposed Indicators and Scoring	Data Source Timing and Conceptual Support
Continuity C Process exposure	Audit information received by the next team, agreed care responsibility, and access to a familiar clinician or team. Score each domain 0–100 using prespecified criteria; average with equal weights and report components and missingness.	Record and handoff audit on days 1–7. Candidate formative index. Continuity definitions [4,14]; patient-reported comparator [17].
Accommodation A Moderator	For baseline needs, verify language support, transport coordination, flexible scheduling, and accessible contact. Score 100 × supported applicable domains/applicable domains. Code no identified need separately; retain uptake separately.	Independent baseline audit matched to patient needs. Candidate formative index. Conceptual support from workload-capacity reasoning and responsive care [6,10,11]. Moderator role and scoring are proposed here.
Navigation burden B Mediator	Original candidate items assess difficulty arranging care, obtaining needed information, and reconciling instructions. Rating: 0—none to 4—extreme; mean × 25 gives 0–100. Record task time and caregiver work separately.	Patient report for days 8–14 and diary corroboration. Validate before confirmatory use. Task/workload foundations [5,9,10]; treatment-burden comparators [18,19].
Patient experience E Intermediate outcome	Original prompts address understandable explanations, respectful treatment, and participation. Rate frequency: 0—never to 4—always; equal domain mean × 25. Report domains separately; exclude continuity, scheduling, and satisfaction items.	Day 21, recalling days 15–21. Candidate composite; analyze whether contact occurred separately. Concept distinction and dimensions [1,6,11].
Patient satisfaction S Evaluative outcome	Original global item assesses overall satisfaction across the transition: 0—not at all satisfied to 10—completely satisfied. Retain the distribution; no assumed threshold for clinical benefit.	Day 30 episode evaluation. Concept and measurement rationale [1,2,12]. This item and outcome role are proposed, not a validated instrument.
Baseline expectations and resources Covariates	Measure expected communication, respect, and involvement; prior experience and satisfaction; clinical complexity, utilization, financial strain, language, disability, digital access, transport, and caregiver support.	Baseline report and records. Prespecify coding. Expectations are not a performance target. Expectations and workload-capacity rationale [5,9,10,12].
Health equity Distributional assessment	Report subgroup levels, changes, and gaps in burden, experience, and needed follow-up completion. Stratify by self-identified social characteristics and practical constraints; no patient-level equity total.	Prespecified comparisons with uncertainty intervals and privacy protection. Equity and social-driver rationale [6,20]. Reporting rules are proposed here.
Health-service quality Balancing outcomes	Track unmet follow-up need, safety events, unplanned acute care, and patient, caregiver, and staff time. Define clinically relevant indicators for the transition pathway before evaluation.	Records and patient/staff reports through day 30. Quality domains and interpretation [3,8,21]. Satisfaction alone is insufficient.

Note. Citations support definitions and theoretical rationale. The roles, candidate items, timing, and scoring rules are proposed in this article and require validation; citations do not validate these new measures. Existing instruments are comparators and should retain validated wording and recall periods unless an adaptation is evaluated.

**Table 2:** Proposed estimands and disconfirming evidence.

Test	Proposed Association or Comparison	Expected Pattern and Disconfirmation
H1	Adjusted C → B slope; estimate at mean A among patients with needs and separately in the broader cohort.	Negative. A precisely null or positive slope does not support H1.
H2	Adjusted B → E slope among patients with assessable contact; report whether contact occurred separately.	Negative. A precisely null or positive slope does not support H2.
H3	Adjusted E → S slope controlling baseline expectations, prior satisfaction, and clinical status.	Positive. A precisely null or negative slope does not support H3.
H4	C × A interaction in the burden model among patients with at least one baseline navigation constraint. Include both main effects.	Negative interaction. A precisely null interaction supports additivity on the specified scale; a positive interaction suggests substitution.
P1	Compare verified remediation with assigned responsibility against acknowledgment alone. Outcome is recurrent navigation failures.	Greater reduction with verified remediation. Similar or worse recurrence challenges P1.
P2	Compare support allocated according to need with uniformly allocated support under equal budgets; examine experience gaps and each group’s level.	Greater gap reduction with improvement in the worse-off group under allocation by need. No such advantage challenges P2.

Note. An estimand is the specific association or comparison a study seeks to estimate. H denotes a patient-level hypothesis and P an organizational proposition. A precisely null result has an interval excluding the prespecified smallest meaningful effect; a nonsignificant *p*-value alone does not establish absence. The conditional indirect association is derived from H1–H4.

## Data Availability

No new participant data were generated or analyzed. The conceptual model and proposed operational definitions are presented in the article, and supporting publications are identified in the reference list.

## Appendix A. Supplementary Search Record

**Table A1:** Exact targeted web queries used during the revision on 12 October 2026.

Topic	Exact Supplementary Web Query	Primary Anchor and Use
Experience and satisfaction	Q1: “patient experience” “patient satisfaction” Bull 2021	Bull [1]; conceptual distinction
Continuity and burden	Q2: “continuity of care” “patient burden” Haggerty May Q3: “continuity of care” Haggerty 2003 “patient burden” May 2014	Haggerty [4]; continuity definitions
Administrative burden	Q4: “patient administrative burden” Kyle Frakt	Kyle and Frakt [5]; navigation and access work
Equity-oriented care	Q5: “equity-oriented care” Browne Varcoe patient experiences 2024	Browne et al. [6]; equity-oriented measurement
Continuity measurement	Q6: “continuity measurement” Ljungholm questionnaire 2024 Q7: Ljungholm “10.1111/jan.15792”	Ljungholm et al. [17]; primary measurement record checked using DOI follow-up
Burden theory	Q8: May “Rethinking the patient” “10.1186/1472-6963-14-281”	May et al. [9]; workload and capacity
Conceptual methods	Q9: Jaakkola “Designing conceptual articles” “four approaches”	Jaakkola [7]; theory synthesis and model development

Note. Queries were entered through the OpenAI web search interface without date filters. Q1–Q9 label separate queries and are not part of the search strings; automatic line wrapping has no syntactic meaning. The first five rows correspond to topics reported in the original manuscript; the final two clarify existing theoretical and methodological anchors. The table records supplementary verification, not the exact searches run on 14 September 2026, a systematic eligibility process, or a count of all web hits. Source numbers identify retained publication anchors, not every returned result. The continuity and measurement rows include follow-up queries because initial broad results are insufficient for primary-source verification. Sources are consulted at the accessible publisher-record, abstract, or full-text level as available; no complete full-text review of every returned source is asserted.
